# Effects of long-term statin-treatment on coronary atherosclerosis in patients with inflammatory joint diseases

**DOI:** 10.1371/journal.pone.0226479

**Published:** 2019-12-12

**Authors:** Mona Svanteson, Silvia Rollefstad, Nils-Einar Kløw, Jonny Hisdal, Eirik Ikdahl, Joseph Sexton, Ylva Haig, Anne Grete Semb

**Affiliations:** 1 Department of Radiology and Nuclear Medicine, Oslo University Hospital, Oslo, Norway; 2 Institute of Clinical Medicine, Faculty of Medicine, University of Oslo, Oslo, Norway; 3 Preventive Cardio-Rheuma Clinic, Department of Rheumatology, Diakonhjemmet Hospital, Oslo, Norway; 4 Department of Vascular Investigations, Oslo University Hospital, Aker, Oslo, Norway; 5 Department of Rheumatology, Diakonhjemmet Hospital, Oslo, Norway; University of Messina, ITALY

## Abstract

**Background:**

The effect of statins over time on coronary atherosclerosis in patients with inflammatory joint diseases (IJD) is unknown. Our aim was to evaluate the change in coronary plaque morphology and volume in long-term statin-treated patients with IJD.

**Methods:**

Sixty-eight patients with IJD and carotid artery plaque(s) underwent coronary computed tomography angiography before and after a mean of 4.7 (range 4.0–6.0) years of statin treatment. The treatment target for low density lipoprotein cholesterol (LDL-c) was ≤1.8 mmol/L. Changes in plaque volume (calcified, mixed/soft and total) and coronary artery calcification (CAC) from baseline to follow-up were assessed using the 17-segment American Heart Association-model.

**Results:**

Median (IQR) increase in CAC after statin treatment was 38 (5–236) Agatston units (p<0.001). Calcified and total plaque volume increased with 5.6 (0.0–49.1) and 2.9 (0.0–23.5) mm^3^, respectively (p<0.001 for both). The median (IQR) change in soft/mixed plaque volume was -10 (-7.1–0.0), p = <0.001. Patients who had obtained the LDL-c treatment target at follow-up, experienced reduced progression of both CAC and total plaque volume compared to patients with LDL-c >1.8mmol/L (21 [2–143] vs. 69 [16–423], p = 0.006 and 0.65 [-1.0–13.9] vs. 13.0 [0.0–60.8] mm^3^, p = 0.019, respectively).

**Conclusions:**

A progression of total atherosclerotic plaque volume in statin-treated patients with IJD was observed. However, soft/mixed plaque volume was reduced, suggesting an alteration in plaque composition. Patients with recommended LDL-c levels at follow-up had reduced atherosclerotic progression compared to patients with LDL-c levels above the treatment target, suggesting a beneficial effect of treatment to guideline-recommended lipid targets in IJD patients.

## Introduction

Patients with inflammatory joint diseases (IJD) have an increased risk of acute coronary syndrome [1]. Lipid-lowering treatment with statins is considered as highly effective prophylaxis for coronary artery disease in the general population due to improvements of both lipid-profiles and clinical outcome [2, 3]. Evidence regarding statin treatment in IJD patients is scarce, but promising results from post hoc analyses in 2 randomized controlled statin trials (TNT and IDEAL) revealed comparable lipid lowering effect and risk reduction for future cardiovascular disease (CVD) in patients with and without IJD [4]. Despite this, inadequate preventive treatment with statins has been reported in patients with IJD [5, 6]. In addition to lowering lipids, statins have been shown to possess anti-inflammatory effects [7]. Other positive plaque-related effects such as cell death in the lipid cores and plaque-stabilization due to micro-calcifications have also been described [8, 9]. Whether these statin effects will occur in patients with IJD is uncertain, due to the underlying systemic inflammation, the lipid increasing effect of anti-rheumatic medications and the polypharmacy these patients have [10]. Inflammation is part of the atherogenesis [11], and elevated inflammation as measured by CRP has been shown be a predictor of increased atherogenesis with clinical outcomes [12]. Assessments of plaque morphology are important and of great interest since non-calcified atherosclerotic plaques are more likely to result in acute coronary syndrome than the more stable calcified plaques [13].

Coronary computed tomography angiography (CCTA) has become an established non-invasive method for detection of coronary artery stenosis [14]. It is also a promising and increasingly used tool for characterization of coronary plaques with good correlation to intravascular ultrasound [15]. Statin-treatment has been shown by CCTA to induce regression of coronary plaques in patients without IJD [16], in addition to a slower progression of coronary plaque volume in patients with low LDL-c level [17]. Increased coronary artery calcifications (CAC) have also been reported after statin treatment in the general population [18]. Taking into consideration that patients with IJD have high systemic inflammation and that disease activity has been shown to have an impact on carotid artery plaque composition [19] further warrants evaluation of the statin effect on atherosclerotic plaques in patients with IJD.

The aims of the present study were to evaluate the progression of coronary atherosclerosis/plaques after long-term statin-treatment in patients with IJD, and the effect on plaque morphology evaluated by CCTA. Furthermore, we assessed possible predictors of plaque progression, including patient characteristics, lipids and inflammatory markers.

## Materials and methods

### Patients and study design

The RORA-AS study (ROsuvastatin in Rheumatoid Arthritis, Ankylosing Spondylitis and other inflammatory joint diseases) was an open, prospective intervention study, and a complete description of inclusion and exclusion-criteria has previously been reported [20]. In short, IJD patients with ultrasound-verified carotid plaque(s) were treated with rosuvastatin with an LDL-c target of ≤1.8 mmol/L, in accordance with the most recent European guidelines [21]. All patients signed an informed consent and the study was approved by the Norwegian South East Regional Committee for Medical and Health Research Ethics and registered with ClinicalTrials.gov Id: NCT01389388. The European Union Drug Regulating Authorities Clinical Trials (EudraCT) number is 2008-005551-20.

CCTA was performed for study purposes in 68 statin-naïve patients with IJD and carotid artery plaques between 2010 and 2012 with a follow-up CCTA in 2016. The follow-up time was prolonged compared to study protocol, due to lack of time available on the scanner. Patients with reduced kidney function (estimated glomerular filtration rate of <45 ml/minute), arrhythmias, previous coronary artery bypass surgery, stents or pacemaker-implantation were excluded. All patients filled in a questionnaire at baseline and follow-up for assessment of characteristics, symptoms of coronary disease and medications. Changes in lipid-profiles and inflammatory parameters were evaluated by laboratory tests drawn and analyzed at Diakonhjemmet Hospital using a COBAS 6000 and COBAS 8000, Roche Diagnostics Norway AS.

### Medications

After baseline CCTA, all patients received rosuvastatin, with dose titration to achieve an LDL-c goal of ≤1.8mmol/L. The lipids were frequently monitored for the first 18 months. Due to national regulations the lipid lowering medication was switched to atorvastatin after the first 18 months unless there was a specific reason to continue rosuvastatin treatment, such as side effects or inadequate lipid lowering effect with other statins. After 18 months the patient was followed by the primary care physician who had received a discharge report including specification of diagnosis, present medication use, LDL-c goal and follow-up recommendations.

### Imaging technique

All baseline and follow-up CCTA examinations were performed on a Philips Brilliance 64-slice CT scanner (Philips Healthcare, Cleveland, Ohio, USA) with protocols as previously described [22]. Initially, a non-contrast scan was conducted for evaluation of CAC. If tolerated, intravenous beta blockage (5–20 mg Seloken®, Astra Zeneca) was used to reduce the heart rhythm and Nitroglycerin 0.4mg (Nitrolingual®, Pohl-Boskamp, Hohenlockstedt, Germany) was administered for the vasodilating effect sublingually 1–3 minutes prior to the contrast-enhanced scan. Prospective ECG-gating was used when achieving a heart rate ≤ 65 beats/min (bpm), while retrospective ECG-gating was required for higher heartrates. The contrast media Omnipaque^TM^ 350 mg/ml (GE Healthcare, Princeton, New Jersey) was used in both the baseline and follow-up examinations.

### Image analysis

The image analyses were performed on a Philips Workstation (Intellispace v5, Philips Healthcare) with dedicated software (Plaque Analysis, Comprehensive Cardiac, Philips Healthcare) [23]. The inter-observer variability was calculated on a per-segment level after two independent readers blinded to patient characteristics measured the plaque volume in left ascending artery in 30% of the patients, with an interclass correlation coefficient 0.92. The same segments were evaluated twice by one reader with an intra-observer variability of 0.93. The analyses were assessed using the 17-segment model of the American Heart Association [24]. All segments with sufficient image quality and a diameter >1.5 mm were included in the analyses.

CAC was calculated by the Agatston method [25]. The morphology of the plaques was defined according to plaque density, measured with Hounsfield Units (HU). Plaques were defined as calcified if ≥90% of the total volume had a density ≥130 HU, and soft when ≥10% had a density of ≥130HU. Mixed plaques were all in between [23]. Coronary artery disease (CAD) was defined as “presence of any plaque.” Segment involvement score (SIS) and segment stenosis score was used to assess extent and severity of the CAD with previously described definitions [22].

### Statistical analysis

Descriptive data are presented with number (%) for dichotomized variables, mean±standard deviation (SD) for normally distributed characteristics or median with interquartile range (IQR) if not normally distributed. Analysis of variance and X^2^ were used to compare variables between groups. The paired samples t-test was applied in assessment of changes in variables from baseline to follow-up. The Wilcoxon signed rank test was used for non-normally distributed variables.

Independent samples t-test was used to test the difference in atherosclerotic change between patients with obtained and non-obtained LDL-c goal at follow-up. Non-normally distributed variables were log-transformed before these analyses were conducted.

Linear regression models were constructed with a stepwise backwards approach to assess predictors of change in plaque volumes and CAC. Pearsons correlation coefficient was used to test correlation to variables (patient characteristics and CVD risk factors), but due to a correlation less than 0.2, none of the variables tested in the univariate analyses were included in the multivariate models. Variables considered to be of clinical relevance (lipids, inflammatory markers and sDMARDS/dDMARDS) were therefore the only variables included in the models.

For further evaluation of atherosclerotic progression, we arbitrary divided the change in total plaque volume into percentiles (25%, 50% and 75%). Differences were evaluated with analysis of variance. Multiple logistic regression was used to identify predictors for <25^th^ percentile and >75^th^ percentile. All analyses were performed using IBM SPSS version 21.

## Results

**Fig 1:**
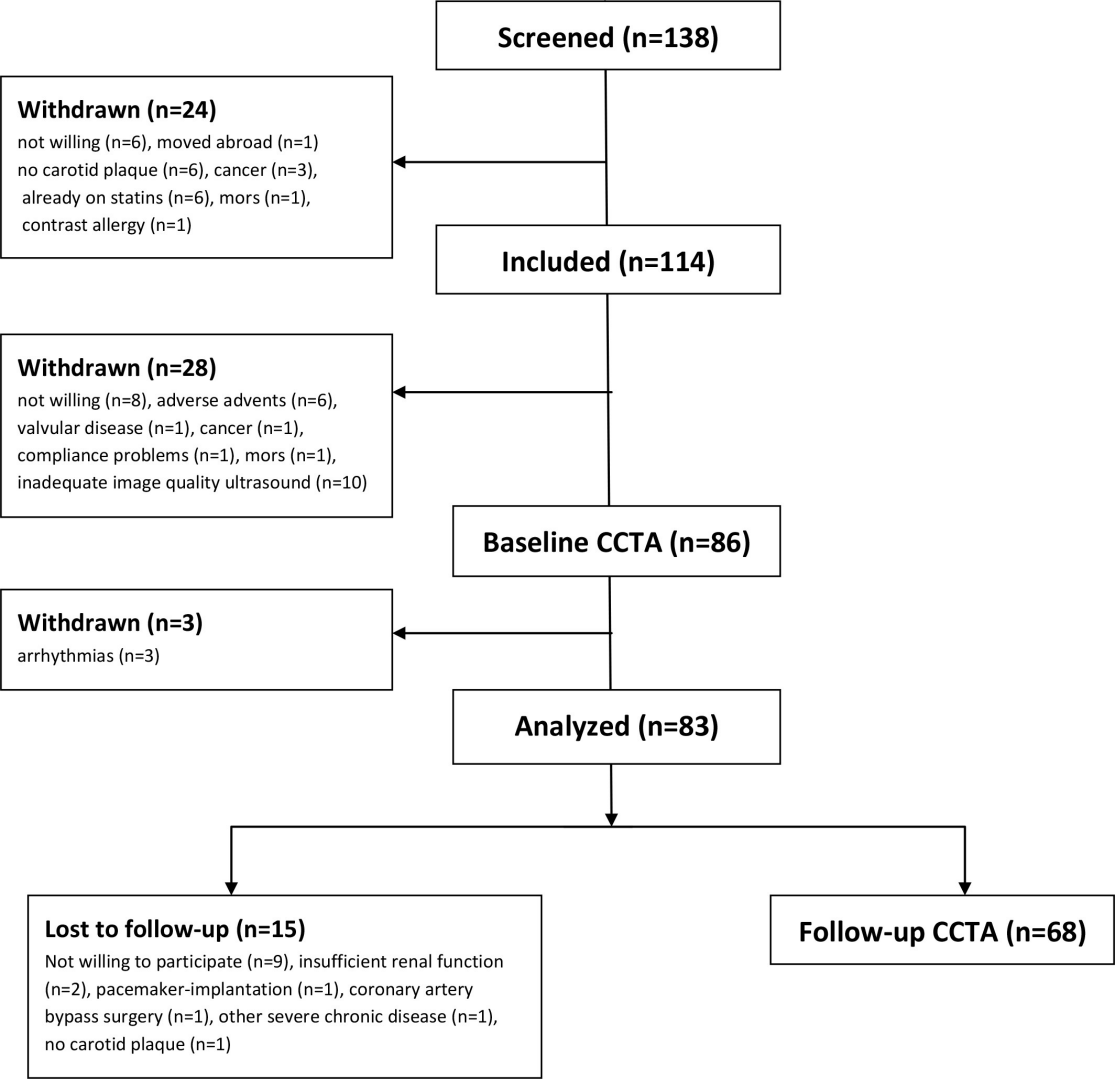
Study flow chart. Out of 83 patients analyzed at baseline CCTA, 68 patients were included at follow-up CCTA.

Of the 83 patients initially included at baseline, 15 patients were lost to follow up; 2 due to insufficient renal function, 1 due to pacemaker-implantation, 1 had a coronary artery bypass surgery, 1 due to severe chronic disease, 1 because of screening failure (no presence of carotid artery plaque at baseline) and 9 did not want to participate (Fig 1). Evaluations from the remaining 68 patients are included in the analyses. Mean follow-up time was 4.7 (range 4.0–6.0) years.

Table 1 presents the patient characteristics at baseline. Two-thirds of the patients had RA (66%), and the majority of these patients were females (64%). Mean age was 60.5±8.6 years. Only a few patients had diabetes mellitus (6%) or previous CVD (10%), but other risk factors of CVD were prevalent; hypertension (47%), hyperlipidemia (64%) and smoking (22%).

**Table 1:** Patient characteristics at baseline.

	IJD n = 68 (100)	RA n = 45 (66.1)	AS n = 15 (22.1)	PsA n = 8 (11.8)
Age (years), mean±SD	60.5±8.6	61.2±8.8	60.3±8.2	57.4±8.6
Women, n(%)	44 (63.8)	34 (73.9)	7 (46.7)	3 (37.5)
Disease duration (years), median (IQR)	17.1±11.9	15.8±1.7	22.7±2.7	13.6±5.0
BMI (kg/m^2^), mean±SD	25.1±3.0	25.1±3.1	24.5±2.2	25.8±3.6
Waist circumference (cm), mean±SD	91±11	91±11	90±9.0	94±11
Systolic BP (mmHg) mean±SD	142±20	141±21	144±13	146±28
Diastolic BP(mmHg), mean±SD	83±9	83±9	83±7	86±12
HT,n(%)	32 (47.1)	21 (46.7)	8 (27.6)	3 (37.5)
Diabetes mellitus, n(%)	4 (5.8)	3 (6.5)	1 (6.7)	0 (0.0)
Smoking, n(%)	15 (21.7)	11 (23.9)	2 (13.3)	2 (25.0)
Family history of CVD, n(%)	11 (15.9)	7 (15.6)	1 (6.7)	3 (37.5)
Previous CVD, n(%)	7 (10.3)	5 (11.1)	2 (13.3)	0 (0.0)
Angina, n(%)	12 (17.4)	10 (21.7)	2 (13.3)	0 (0.0)
Hyperlipidemia, n(%)	44 (63.8)	27 (58.7)	4 (73.3)	6 (75.0)
**Medications**				
Synthetic DMARDs, n(%)	40 (62.5)	28 (65.1)	4 (30.8)	8 (100.0)
Biologic DMARDs, n(%)	22 (34.4)	13 (32.5)	5 (38.5)	4 (50.0)
NSAIDs, n(%)	19 (32.8)	13 (32.5)	4 (30.8)	2 (10.5)
Anti-hypertensives, n(%)	10 (14.7)	6 (15.4)	3 (33.3)	1 (16.7)
**Inflammatory markers**				
ESR (mm/hour), mean±SD	11.8±9.3	13.0±10.5	8.4±4.8	9.3±3.6
CRP (mg/L), mean±SD	3.6±4.7	4.0±5.1	2.6±3.6	2.8±2.9

IJD: inflammatory joint disease, RA: rheumatoid arthritis, AS: ankylosing spondylitis, PsA: psoriatric arthritis, BMI: body mass index, BP: blood pressure, HT: hypertension, CVD: cardiovascular disease, DMARDS: disease modifying anti-rheumatic drug, NSAIDs: Non-steroidal Anti-Inflammatory Drugs, ESR: erythrocyte sedimentation rate, CRP: C-reactive protein.

Hyperlipidemia: total cholesterol ≥6.0mmol/l.

Hypertension: systolic BP >140 mmHg and diastolic BP >90 mmHg.

CAD was detected in 42 (62%) patients at baseline, compared to 51 (75%) at follow-up. In total, atherosclerotic plaques were present in 133 of 913 (14.6%) segments at baseline compared to 203 of 874 (23.2%) at follow-up. Forty-six (34.6%) of the plaques were defined as mixed or soft at baseline compared to 16 (7.9%) at follow-up.

The atherosclerotic progression is shown in Table 2.

**Table 2:** CCTA findings, lipids and inflammatory markers at baseline and follow-up (per-patient-level).

	Baseline (n = 68)	Follow-up (n = 68)	Change (n = 68)	p-value
**CCTA findings**				
CAC, Agatston units, median(IQR)	15(0–221)	73(6–514)	38 (5–236)	<0.001_**a**_
Total plaque volume, mm^3^, median(IQR)	5.1(0.0–36.7)	8.0(0.5–77.2)	2.9 (0.0–23.5)	<0.001_**a**_
Calcified plaque volume, mm^3^, median(IQR)	0.2(0.0–15.5)	9.5(6.0–77.2)	5.6 (0.0–49.1)	<0.001_**a**_
Mixed/soft plaque volume, mm^3^, median(IQR)	0(0–8)	0(0–0)	-10 (-7.1–0.0)	0.001_**a**_
Segment Involvement Score	2.0±2.5	3.1±2.9	1.1±1.4	<0.001_**b**_
Segment Stenosis Score	2.9±4.0	5.7±6.3	2.8±3.1	<0.001 _**b**_
**Lipids**				
Total cholesterol, mmol/L	6.44±1.09	4.34±0.85	-2.09±1.14	<0.001_**b**_
HDL cholesterol, mmol/L	1.75±0.55	1.81±0.61	0.07±0.31	0.059_**b**_
LDL cholesterol, mmol/L	4.02±1.02	1.97±0.70	-2.06±1.09	<0.001_**b**_
Triglycerides, mmol/L	1.52±0.98	1.27±0.80	-0.24±0.82	0.019_**b**_
**Inflammation-markers**				
ESR, mm/hour	13.71±9.17	11.88±11.93	-1.83±12.55	0.24_**b**_
CRP, mg/L	3.71±3.86	3.70±5.76	-0.01±6.58	0.99_**b**_

Values are presented as the mean ± SD unless otherwise stated.

_a_Wilcoxon signed rank test

_**b**_Paired samples t-test

* coefficient of variation: 4.3%

CCTA: coronary computed tomography angiography, CAC: coronary artery calcification, SD: standard deviation, HDL: high density lipoprotein, LDL: low density lipoprotein, ESR: erythrocyte sedimentation rate, CRP: C-reactive protein

Median (IQR) increase in CAC increase was 38(5–236) Agatston units (p<0.001). Calcified and total plaque volume increased with 5.6 (0.0–49.1) and 2.9(0.0–23.5) mm3, respectively (p<0.001 for both). The median (IQR) change in soft/mixed plaque volume was -10 (-7.1–0.0), p = <0.001. Regarding lipids, all levels were reduced except for high density lipoprotein cholesterol (HDL-c), as expected. The inflammatory markers were comparable at baseline and follow-up. Both segment involvement score and segment stenosis score increased (p<0.001 for both).

**Fig 2:**
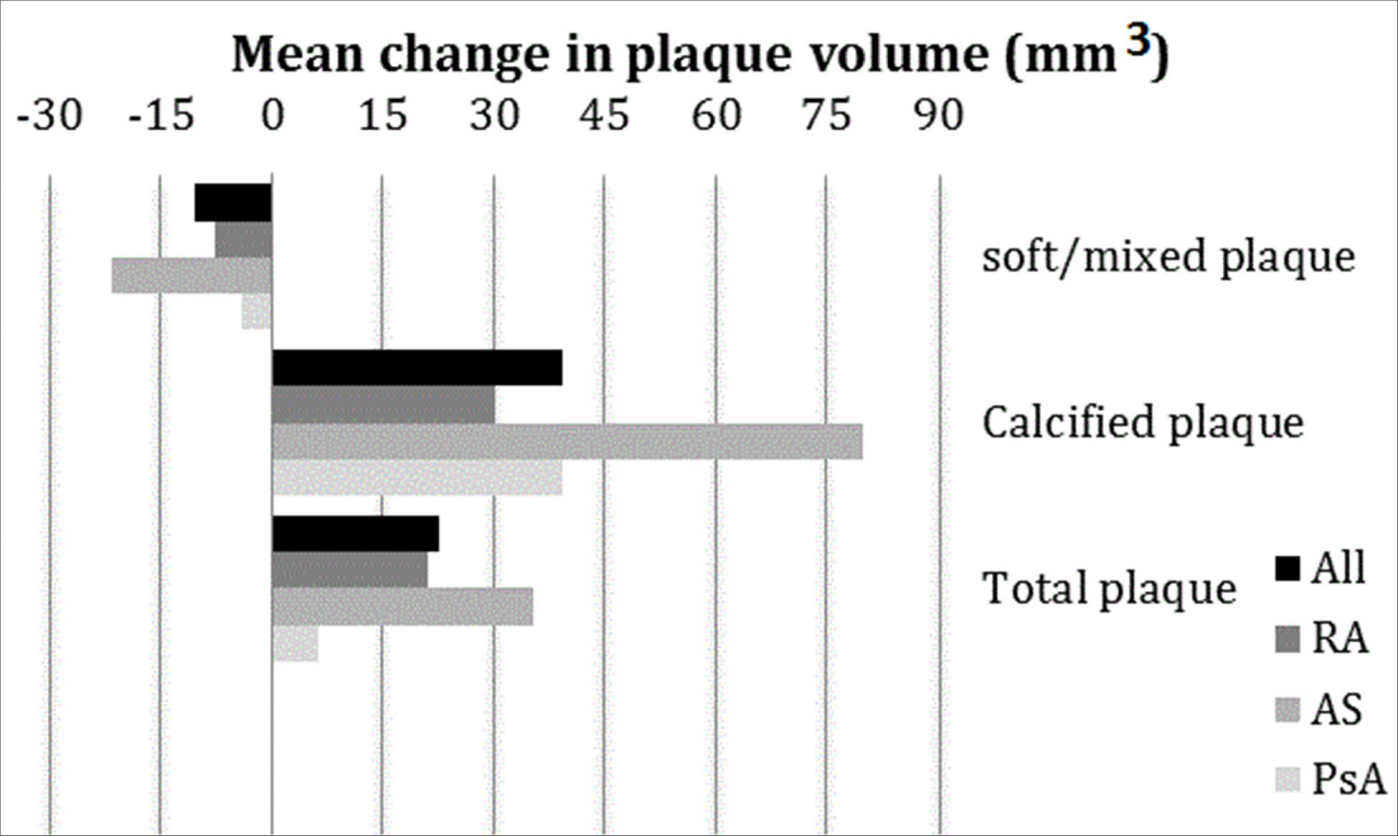
Mean change in plaque volume in the 3 IJD groups. Data shown as mean change in soft/mixed, calcified and total plaque volume (mm^3^). The soft/mixed plaque was over-all reduced, and calcified and total plaque volume increased in all groups. The plaque alterations are highest in the AS-group. RA:rheumatoid arthritis, AS:ankylosing spondylitis, PsA:psoriatic arthritis.

Fig 2 shows the mean change in plaque volume in the 3 IJD groups. The ankylosing spondylitis (AS) group had a larger reduction in soft/mixed plaque volume, and more extensive increase in calcified and total plaque volume than RA and psoriatic arthritis (PsA) patients.

At follow-up, 34 (50%) of the patients had an LDL-c level below study target (≤1.8mmol/L). Table 3 shows the difference in the CCTA-measurements between patients with an LDL-c level above or below 1.8mmol/l at follow-up. The change in CAC, calcified plaque volume and total plaque volume was reduced in the group with an LDL-c ≤1.8 mmol/L. The reduction in soft/mixed plaque volume was numerically larger in the group with LDL-c-level above treatment target, although this difference was not statistically significant (p = 0.71).

**Table 3:** Lipid status and CAD-progression in patients with and not with LDL-c ≤ 1.8mmol/l.

	LDL ≤1.8mmol/l n = 34	LDL >1.8mmol/l n = 34	p-value
**LDL-c level Baseline,** mmol/L, mean±SD	3.7±0.9	4.4±1.0	<0.001
**LDL-c level Follow-up**, mmol/L, mean±SD	1.5±0.2	2.4±0.7	<0.001
**Change LDL-c level**, mmol/L, mean±SD	-2.2±0.9	-1.9±1.3	0.38
**Change CAC**, median (IQR)	21 (2–143)	69 (16–423)	<0.001_a_
**Change Soft/Mixed plaque**, mm^3^, median (IQR)	0 (-3.5–0.0)	0 (-15.7–0.0)	0.71_a_
**Change calcified plaque**, mm^3^, median (IQR)	1.7 (0.0–17.3)	13.4 (1.5–107.6)	<0.019_a_
**Change Total Plaque**, mm^3^, median (IQR)	0.65 (-1.0–13.9)	13.0 (0.0–60.8)	<0.001_a_

_a_independent samples t-test using log-transformed variables.

CAD: coronary artery disease, LDL-c: low density lipoprotein cholesterol, SD: standard deviation, CAC: coronary artery calcifications, IQR: interquartile range

An LDL-c level >1.8mmol/l was associated with change in CAC (model A) and change in total plaque volume (model B), after adjusting for age and sex, but was not significantly associated with change in soft/mixed plaque volume (model C) (Table 4).

**Table 4:** Associations between progression of CAC (A), total plaque volume (B), soft/mixed plaque volume (C) with lipids, inflammatory markers and sDMARDS/bDMARDS.

		Univariate		Multivariate^d^	
		β (95%CI)	p-value	β (95%CI)	p-value
**A**_a_	Age	5.62 (-2.51-13-75)	0.17	8.80 (1.14–16.45)	0.025
	Male	57.21 (-86.39- -200.80)	0.43	79.25 (-52.69–211.19)	0.23
	LDL-c >1.8mmol/l	199.63 (69.09–330.16)	<0.001	225.79 (95.48–356.09)	0.001
	HDL-c	49.90 (-164.97–264.75)	0.64		
	Triglycerides	47.11 (-38.40–132.62)	0.28		
	CRP follow-up	-2.85 (-15.03–9.34)	0.64		
	ESR follow-up	0.67 (-5.48–6.83)	0.83		
	Non-bDMARDs user	70.39 (82.99–223.77)	0.36		
	Non-sDMARDs user	122.13 (26.22–270.47)	0.11		
**B**_**b**_	Age	0.57 (-0.97–2.12)	0.46	0.97 (-0.53–2.47)	0.20
	Male	21.87(-5.03–48.76)	0.11	23.5 (-2.68–49.80)	0.078
	LDLc >1.8mmol/L	30.42 (5.06–55.79)	0.019	33.8 (8.2–59.4)	0.010
**C**_**c**_	Age	-0.37 (-1.15–0.41)	0.35	-0.48 (-1.24–0.27)	0.21
	Male	-18.06 (-31.26- -4.85)	<0.001	-18.05 (-31.25- -4.84)	0.008
	LDL-c >1.8mmol/L	-9.15 (-22.39–4.09)	0.17	-11.52 (-24.41–1.37)	0.079

Linear regression A_a_: change in CAC as dependent variable, B_b_: change in total plaque volume, C_c_: change in soft/mixed plaque volume

^d^Adjusted for number of months between baseline and follow-up.

CAC: coronary artery calcification, CI: confidence interval, LDL: low-density lipoprotein, sDMARDs: synthetic disease modifying anti-rheumatic drugs, bMARDS: biologic disease modifying anti-rheumatic drugs, CRP: C-reactive protein, ESR: erythrocyte sedimentation rate.

S1 Fig shows a near linear relationship between change in total mixed/soft plaque volume per patient and baseline mixed/soft plaque volume (R = 0.898).

**Fig 3:**
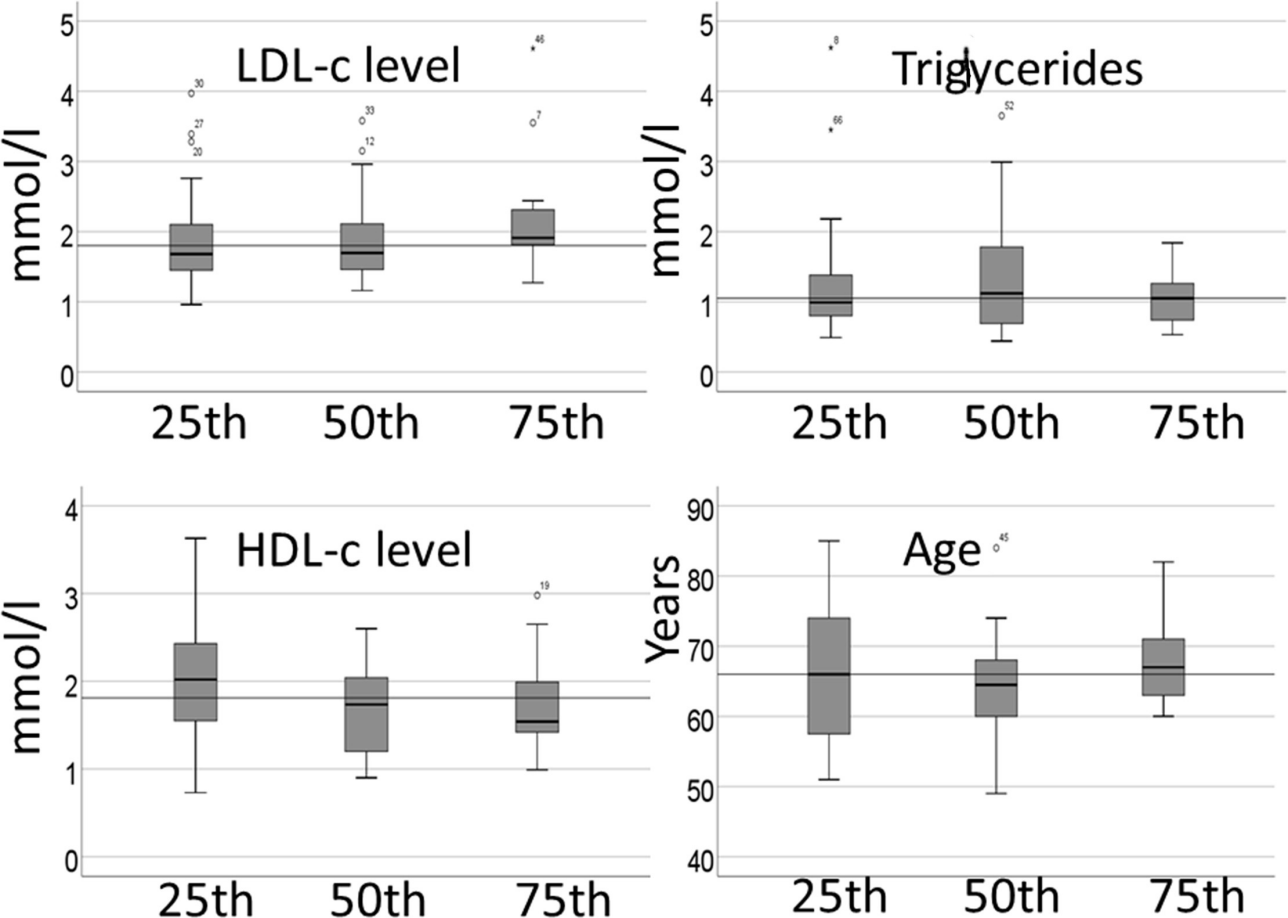
Difference in lipids and age between percentiles of increase in total plaque volume (mm^3^). The reference line is set to median in all variables. LDL-c; low density lipoprotein-cholesterol, HDL-c; high density lipoprotein-cholesterol.

Fig 3 presents the difference in HDL-c, LDL-c, triglycerides and age between the percentiles of change in total plaque volume, with no significant difference between the groups. However, in the multiple logistic regression analysis, the HDL-c level at follow up was associated with <25^th^ percentile (<2.9 mm^3^) increase in total plaque volume, OR (95%CI): 3.36 (1.16–9.74), p = 0.029, after adjusting for age and sex. In addition, LDL-c (OR: (95%CI): 1.3 (1.2–11.0), p = 0.022) was associated with the >75^th^ percentile (>23.5mm^3^) of change in total plaque volume after adjusting for sex and age. All patients with ≥400 CAC increase had an LDL-c-level at follow-up above the treatment target.

The correlation between biologic DMARD-use and change in CAC, total plaque volume, soft/mixed plaque volume and calcified volume were: r = -0.14 (p = 0.28), r = 0.12 (p = 0.36), r = 0.03 (p = 0.81) and r = -0.02 (p = 0.88), respectively.

## Discussion

In this study, we have shown that a progression of coronary atherosclerosis in statin-treated patients with IJD occurs after nearly 5 years of statin treatment. However, an increase in calcified plaque volume and a decrease in soft/mixed plaque volume suggested a conversion in plaque-composition. We also revealed that LDL-c-levels were associated with atherosclerotic progression in the sense that the patients who obtained LDL-c treatment target experienced a more moderate progression of atherosclerotic plaque volume compared to those with LDL-c-levels above the LDL-c treatment target of 1.8 mmol/L. To our knowledge, this is the first study to assess the effects of statin-treatment on coronary plaques in patients with IJD.

The CAC increased significantly from baseline to follow-up. CAC has a well-documented prognostic value for future cardiac events, and a linear relationship between CAC and CVD risk has been established [26–28]. CAC has been shown to be a greater determinant of atherosclerotic progression than traditional risk-factors, sex or age in asymptomatic individuals [29]. However, the relationship of CAC progression and events has not been fully elucidated in statin users [30]. Puri *et al*. described that an increase in CAC induced by statins had a positive plaque-stabilizing effect due to induction of micro-calcifications [8]. Shaw et al. suggested that CAC may loose its predictive value after initiation of plaque-altering therapies such as statins [30]. From the MESA-study [31] it was reported an inverse association between plaque density and risk of CVD events, suggesting that denser plaques may be protective for CVD events. Whether the increased CAC in our study was a marker for healing of plaques (induced by statins) or for progression of disease, is difficult to interpret. However, the volume measurements add valuable information to this evaluation, as the total plaque volume also increased significantly in our study. If the increased CAC was solely due to statin-treatment, the volume may not increase significantly and thus, one may argue that the CAC increase in our study is, most likely, an effect caused by both plaque-stabilizing and disease progression.

Another important finding is the reduction in mixed/soft plaques from baseline to follow-up. The presence of soft plaques has been reported to be an independent predictor for acute coronary syndromes [32], and a reduction of soft/mixed plaque is likely to be beneficial for the patient. Previous studies have reported on a difference in plaque morphology between statin users and non-statin users [33]. Further, statins have shown a greater impact on the morphology of non-calcified/partially calcified plaques than on solely calcified plaques [34].

Interestingly, we observed a significantly lower progression of both CAC and plaque volume among the patients who maintained LDL-c-levels of ≤1.8 mmol/l at follow-up. The latter finding is in line with results from a 10-year follow-up study by Goh *et al*., showing a slower progression of CAC in patients on aggressive statin treatment regimens [35]. Two other studies have found reduced progression of plaque volume additionally to CAC in patients who achieved lower LDL-c-levels [17, 36]. Zeb *et*. *al* found a slower progression in non-calcified atheroma after 1 year follow-up in statin-users compared to non-statin-users [37]. A recently published study, described a significant association between individual lipoprotein variability and coronary atheroma progression and also to adverse CVD events [38]. We did not manage to detect a significant difference in regression/progression in soft/mixed plaque volume in those with an LDL-c level above vs. below the LDL-c treatment target. However, there was a near linear relationship between the regression of the volume of mixed/soft plaque and mixed/soft plaque at baseline (S1 Fig). Thus, the group with the largest burden of soft/mixed plaques at baseline experienced most regression/alteration (i.e. those with LDL-c >1.8mmol/l). This finding might be influenced by the “regression towards the mean-“phenomenon. However; the number of soft/mixed plaques was also significantly reduced. Fig 2 shows more plaque alterations/regression of soft/mixed plaque in the AS-group compared to the RA and PsA groups. The AS-group consisted of more males in comparison with the RA and PsA groups, which may explain the higher presence of more soft/mixed plaque at baseline.

In our study, LDL-c and HDL-c-levels in addition to age turned out as important predictors of atherosclerotic progression. The significant association between LDL-c level and progression of both CAC and total plaque volume was maintained after adjusting for sex and age in multivariate analyses. Along the same lines, the LDL-c level was predictive of the patient ending up with a total plaque volume above the 75^th^ percentile, suggesting that the LDL-c level also plays an important role in plaque progression in patients with IJD. Moreover, a higher HDL-c-level was a predictor for having a small increase in total plaque volume (<25^th^ percentile). This finding is consistent with previous reports on the protective effect of HDL-c on atherosclerosis [39].

Atherosclerosis is a multifactorial and complex disease in which inflammation has been shown to play an important role. The pleiotropic effect of statins has shown to also reduce the inflammation markers [7], which may be beneficial in patients with systemic inflammation. In our study, both ESR and CRP were not significantly reduced from baseline to follow-up, and neither was related to plaque progression/regression during the follow up period of 4.7 years. Furthermore, we did not find an association between markers of inflammatory disease activity at baseline and progression of CAD. The latter is probably due to the fact that the patient cohort was well treated with anti-inflammatory drugs when entering the study (mostly in remission or with low disease activity). The lack of association between CAD and inflammatory markers in our study may therefor suffer from a type II error, as we may not have sufficient variations in these variables to detect statistically significant associations. We cannot exclude a type II error also in the negative associations to biologic DMARDS in present study. Only 13 patients were on biologic DMARDS which may have resulted in lack of power.

A clear limitation to our study is the absence of a placebo controlled arm of non-statin users, which would have been helpful in identification of plaque progression/regression caused by statins, especially the reported statin-effect on CAC progression. Furthermore, the loss of 15 patients to follow-up may have influenced our results, as the progression of atherosclerosis in these patients is unknown.

A recently published systematic review implies that CCTA has a potential role in assessment on the response of statin therapy on plaque volume and composition [40]. Such serial plaque assessments demand usage of the same software [41, 42]. In our study, plaque assessments were performed with a software previously shown to have a high degree of inter-observer variability on calcified and mixed lesions [23]. However; overestimation of calcified plaques due to blooming artifacts is a known limitation in CCTA [43]. Therefore we also evaluated CAC-score and number of plaques, with comparable results as with the volume-measurements. CAC is an established method with a high degree of reproducibility [44]. Importantly, the observer variability in our study was shown to be smaller than the actual change in plaque burden when comparing serial CT examinations [45].

After the 18 months follow-up in the study, the patients’ cardiovascular preventive care was transferred to the primary care physician, who was responsible for further management of the statin-treatment. A lack of control of the medicine intake and lipid-levels in the period between 1.5 and 4.7 years may have influenced our results as we have not measured sequential LDL-c levels at regular intervals during this period. However; we believe it is of clinical importance to evaluate the development of plaque progression and lipid profiles in a real-life, clinical setting. Interestingly, 50% of the patients maintained the LDL-c treatment target of ≤ 1.8 mmol/L during the follow up time, which is higher than reported from the general population [46].

In conclusion, we revealed a progression of atherosclerotic plaque volume in statin-treated patients with IJD. However, after long-term statin treatment the number of soft, unstable plaques was reduced, and the calcified plaques were more abundant. An explanation for this may be that statin treatment induced an alteration in plaque composition from mixed/soft plaques into calcified plaques in patients with IJD. Patients with recommended LDL-c levels below 1.8 mmol/L after nearly 5 years of statin-treatment, experienced a reduced atherosclerotic progression compared to patients with LDL-c levels above this treatment target. Our results support the importance of treatment to guideline recommended lipid targets in IJD patients. Longitudinal studies for assessment of the effect of statins and plaque morphology on CVD events in IJD patients are warranted.

## Supporting information

S1 File The ROsuvastatin in Rheumatoid Arthritis, Ankylosing Spondylitis and other inflammatory joint diseases (RORA-AS) Study Protocol in English. (PDF)

S2 File The ROsuvastatin in Rheumatoid Arthritis, Ankylosing Spondylitis and other inflammatory joint diseases (RORA-AS) Study Protocol in original language (Norwegian). (PDF)

S3 File The Transparent Reporting of Evaluations with Nonrandomized Designs (TREND) checklist for the RORA-AS study. (PDF)

S1 Fig Change in soft/mixed plaque volume in relation to baseline soft/mixed plaque volume. A linear relationship between baseline soft/mixed plaque volume and change in soft/mixed plaque volume was detected (R = 0.898, p<0.001).
(TIF)
