# Supplementary figures and images for: Effects of long-term statin-treatment on coronary atherosclerosis in patients with inflammatory joint diseases
Source: PLoS One. 2019 Dec 12;14(12):e0226479. doi: 10.1371/journal.pone.0226479 (PMC6908439; doi:10.1371/journal.pone.0226479)

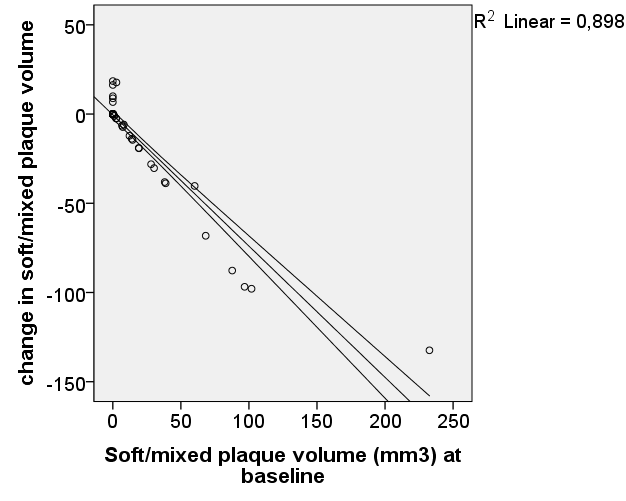

Supplement: S1 Fig — A linear relationship between baseline soft/mixed plaque volume and change in soft/mixed plaque volume was detected (R = 0.898, p<0.001). (TIF) [file pone.0226479.s004.tif]
